# Transforming Growth Factor-Beta and Oxidative Stress Interplay: Implications in Tumorigenesis and Cancer Progression

**DOI:** 10.1155/2015/654594

**Published:** 2015-05-20

**Authors:** Jelena Krstić, Drenka Trivanović, Slavko Mojsilović, Juan F. Santibanez

**Affiliations:** Laboratory for Experimental Hematology and Stem Cells, Institute for Medical Research, University of Belgrade, Dr. Subotića 4, 11129 Belgrade, Serbia

## Abstract

Transforming growth factor-beta (TGF-*β*) and oxidative stress/Reactive Oxygen Species (ROS) both have pivotal roles in health and disease. In this review we are analyzing the interplay between TGF-*β* and ROS in tumorigenesis and cancer progression. They have contradictory roles in cancer progression since both can have antitumor effects, through the induction of cell death, senescence and cell cycle arrest, and protumor effects by contributing to cancer cell spreading, proliferation, survival, and metastasis. TGF-*β* can control ROS production directly or by downregulating antioxidative systems. Meanwhile, ROS can influence TGF-*β* signaling and increase its expression as well as its activation from the latent complex. This way, both are building a strong interplay which can be taken as an advantage by cancer cells in order to increment their malignancy. In addition, both TGF-*β* and ROS are able to induce cell senescence, which in one way protects damaged cells from neoplastic transformation but also may collaborate in cancer progression. The mutual collaboration of TGF-*β* and ROS in tumorigenesis is highly complex, and, due to their differential roles in tumor progression, careful consideration should be taken when thinking of combinatorial targeting in cancer therapies.

## 1. Introduction

Metastasis results from a complex molecular cascade which allows cancer cells to gain malignancy and leave the primary tumor mass and disseminate to distant anatomical sites where they can proliferate and form secondary tumor foci. Disseminated disease is the most usual cause of death in cancer patients and is, therefore, a very serious clinical problem [1]. Transforming growth factor-beta (TGF-*β*) has been postulated to have a dual role in tumor progression, acting as a tumor suppressor in early stages of carcinogenesis and exerting a prooncogenic role in the last steps of the metastatic disease [2]. TGF-*β* induces the epithelial mesenchymal transition (EMT) of transformed cells, which contributes to tumor invasion and metastasis, and is frequently overexpressed in carcinoma cells [3–7].

In normal physiological conditions, Reactive Oxygen Species (ROS) producers constantly generate ROS, while they are eliminated by ROS scavenging systems, thus maintaining redox homeostasis. Redox imbalance, due to aberrant ROS production and/or antioxidant functionality, contributes to tumor progression and is a hallmark of several types of cancer [8, 9]. ROS may participate in cancer initiation, progression, and spreading acting as secondary messengers in the activation and maintenance of signaling pathways which regulate cellular proliferation, survival, angiogenesis, EMT, and metastasis [9]. It is believed that ROS mediate many effects of TGF-*β* during tumorigenesis, since they participate in the regulation of downstream TGF-*β* signal transduction which involves Smads, MAPKs, and NF-*κ*B, as well as the increase of cell motility [10–12]. Meanwhile, TGF-*β* is able to regulate ROS levels by both enhancing their production and reducing antioxidative/scavenging systems activity [10, 13]. Moreover, increased ROS levels in turn may increment TGF-*β* expression and stimulate the release of TGF-*β* from the secreted latent complex making this growth factor bioavailable and active [10, 14].

Both ROS and TGF-*β* have important roles in cellular senescence; ROS can induce cell damage at macromolecular levels, including damage in nucleic acids, a mechanism critical for the development of several age-associated diseases [15]. In turn, TGF-*β* is able to induce senescence in the early stages of epithelial tumorigenesis [2], partly through a mechanism implicating ROS production. Tumor cells can escape senescence by dysregulation in the TGF-*β* signaling. In addition, TGF-*β*, induced by ROS, may affect cancer-associated fibroblast by inducing senescence [16] which in turn collaborates in the enhancement of tumor progression.

Thus, a strong positive feedback between TGF-*β* and oxidative stress/ROS can be established contributing this way to tumor progression. The aim of this review is to reflect on TGF-*β* as a key molecule in cancer and its molecular interplay with the oxidative stress produced by ROS, taking into account that both are involved in the complex cascade of events that culminate in cancer cell metastasis.

## 2. Transforming Growth Factor-Beta

TGF-*β*1 belongs to a large family of more than 40 structurally related regulatory proteins expressed in mammals, which are organized in several subfamilies: TGF-*β*s, bone morphogenetic proteins (BMPs), growth and differentiation factors (GDFs), Mullerian inhibitory factor (MIF), activins, and inhibins [17, 18]. Among the TGF-*β*s, mammals express three genetically distinct isoforms (TGF-*β*1, *β*2, and *β*3) with high homology. The corresponding human genes are located on chromosomes 19q13, 1q41, and 14q24, respectively [19]. In addition to its important role in processes involved in normal development, which include cell growth and differentiation, TGF-*β* is also involved in tumorigenesis.

**Figure 1:**
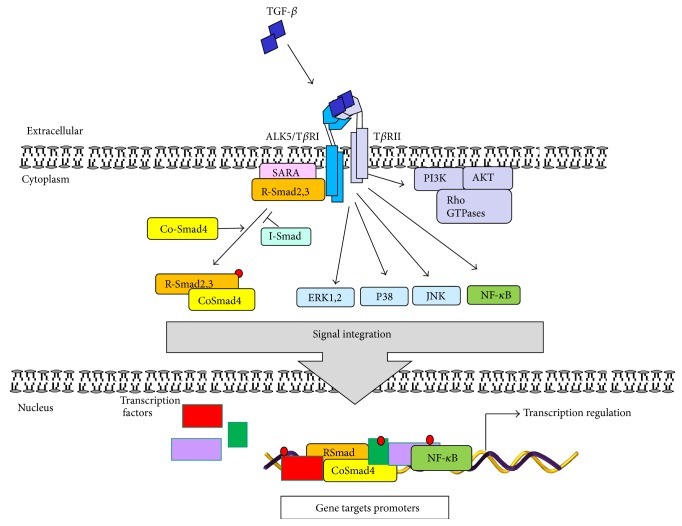
TGF-*β* signaling. Active TGF-*β*1 binds to its cell surface type II receptor (T*β*RII) inducing the activation of TGF-*β* type I receptor (ALK5/T*β*RI) forming a heterotetrameric complex. Active T*β*RI from the complex then triggers the activation of the Smad pathway: T*β*RI phosphorylates the receptor associated-Smads (R-Smads) Smad2,3 which in turn promotes their release from the complex with SARA from the inner face of plasma membrane. Phosphorylated Smads interact with co-Smad4 forming a heteromeric complex to be translocated into the cell nucleus, where, through the interaction with other transcription factors and corepressors or coactivators, it modulates gene expression. Active TGF-*β*-receptors can also activate the non-Smad signaling pathways, such as ERK1,2, p38, JNK, and NF-*κ*B. Furthermore, the active receptor complex can activate PI3K provoking the activation of AKT and the small Rho GTPases. The activation of non-Smad signaling pathways can, in turn, initiate transcriptional or nontranscriptional activity to regulate gene and cellular responses.

The TGF-*β* signaling starts when TGF-*β* dimmer binds to a heteromeric complex made of two cell surface serine/threonine kinase receptors: TGF-*β* type I receptor (T*β*RI/ALK5, activin A receptor type II-like kinase 5) and TGF-*β* type II receptor (T*β*RII). Binding of TGF-*β* dimer to T*β*RII leads to the phosphorylation and activation of T*β*RI/ALK5 [20]. The cytoplasmatic mediators Smad2 and -3 that belong to receptor associated-Smads (R-Smads), a subgroup of Smads, are then phosphorylated by the activated receptor type I, which stimulates the release of Smads from the complex formed with Smad anchor for receptor activation (SARA). Activated Smad2,3 further bind to the common Smad4 (co-Smad4) and are translocated into the nucleus. This Smad complex interacts with several transcription factors, coactivators, or repressors to regulate the expression of different target genes (Figure 1) [19, 20].

TGF-*β* signaling is regulated by the inhibitory Smad proteins (I-Smads), Smad6 and Smad7. Principally, Smad7 antagonizes TGF-*β* by interacting with T*β*RI and leading to its degradation, and Smad6 inhibits the BMP signaling by Smad1–Smad6 complex formation [21]. On the other hand, TGF-*β* signaling regulates I-Smads expression, this way establishing a negative feedback loop. The activity of TGF-*β* and its receptors can also be regulated by the type III nonkinase receptor (T*β*RIII) endoglin or betaglycan which are able to form a complex with other TGF-*β* receptors [22]. In addition, TGF-*β*/TGF-*β* receptor/Smad cascade is subject to posttranslational modification which finely regulates TGF-*β* signaling. These include processes such as phosphorylation/dephosphorylation, sumoylation, and/or ubiquitination which reversibly regulate receptor and Smad stability and availability. Also, ligand-receptor complexes can be internalized and recycled via lipid rafts/caveolae or clathrin coated vesicles and lead to TGF-*β* protein degradation in the proteasome, this way attributing to the modulation of TGF-*β* signaling [23]. TGF-*β* also activates several non-Smad pathways such as the mitogen-activated protein kinases (MAPKs), phosphoinositide 3-kinase (PI3K), rac-alpha serine/threonine-protein kinases (AKT1,2), nuclear factor *κ*B (NF-*κ*B), cyclooxygenase-2, and prostaglandins and the small GTPase proteins: Ras and Rho family (Rho, Rac1, and Cdc42), among others (Figure 1) [24, 25].

## 3. The Role of TGF-***β*** in Cancer 

Depending on the cancer stage, TGF-*β* can operate as a tumor suppressor or as a tumor promoter. Due to its antiproliferative and proapoptotic roles, TGF-*β* protects the injured or stressed epithelium from local mitogenic stimulation in the early stage of epithelial carcinogenesis. During advanced stages of carcinogenesis, cancer cells become resistant to the protective effects of TGF-*β* by different mechanisms, including modifications in the components of TGF-*β* signaling, such as inactivating mutations in T*β*RII and Smad4, and other not fully elucidated alterations [20, 26, 27].

Cancer cells use the capacity of TGF-*β* as the most potent immunosuppressive cytokine, to escape the immune system surveillance and to induce tumor growth and metastasis [28]. In order to do so, cancer cells secrete elevated levels of TGF-*β*, which acts on nontransformed cells present in tumor microenvironment, as well as distant cells in the host, this way suppressing antitumor immune responses and creating an environment of immune tolerance [29, 30]. In addition, TGF-*β* acts as a chemoattractant for monocytes and macrophages, which then migrate toward the tumor. Macrophages modified in tumor microenvironment further support tumor invasion, angiogenesis, and metastasis and also lead to diminished antigen presentation [31].

The importance of the TGF-*β* signaling pathway in human cancers is evident from the frequent alterations of TGF-*β* signaling components in hereditary human cancers and sporadic cancers [32]. Several tumors express high levels of TGF-*β*, correlating with tumor progression and clinical prognosis.

Both cancer cells and local stroma produce TGF-*β*, which, through autocrine and paracrine effects, induces cancer growth and its metastatic potential [30]. Elevated levels of TGF-*β* in plasma have been noticed in a number of cancer types. In breast, prostate, pancreatic, and renal cancer they have been related to advanced cancer stage, metastases, and poor clinical outcome [33–35]. In addition, elevated serum levels of TGF-*β* have been observed in myeloma patients, where both malignant cells and bone marrow stromal cells secrete TGF-*β* [36]. TGF-*β* levels are also elevated in non-Hodgkin's lymphoma and are markedly elevated in high-grade lymphomas, cutaneous T cell lymphomas with a T-regulatory phenotype, and splenic marginal zone lymphomas presenting as myelofibrosis [30].

Several hereditary cancer syndromes with mutations in TGF-*β* superfamily members are known. The autosomal dominant familial juvenile polyposis syndrome (JPS) is the most common of the hamartomatous syndromes [37]. Patients with JPS present multiple hamartomatous polyps in the gastrointestinal tract, predominantly in the colon, at a young age, and predispose individuals to gastrointestinal tract cancers [32]. Germline mutations in different members of the TGF-*β* superfamily have been described in these patients. In 20–25% of cases BMP receptor type IA is mutated, the majority in the kinase domain; 15–20% has* SMAD4* mutations, predominantly in the MH2 domain. Mutations in endoglin gene have also been established, but the incidence is unknown [38, 39].

Homozygous deletion or intragenic inactivating mutations of* SMAD4*, also named* DPC4 *for Deleted in Pancreatic Cancer locus 4, as well as the complete loss of Smad4 protein expression, are observed in 50% ductal adenocarcinomas, 34% of invasive adenocarcinomas of the Vater ampulla and 55% endocrine pancreatic carcinomas [40]. In addition,* SMAD4* is mutated in 50% of all human pancreatic cancers, supporting the important tumor suppressor role of the TGF-*β* pathway [41]. The autosomal dominant disorder hereditary nonpolyposis colorectal cancer (HNPCC) is the most common hereditary predisposition for the development of colorectal cancer. HNPCC is caused by germline mutations involving DNA mismatch repair system genes and contributes to microsatellite instability [42]. T*β*RII gene contains a 10-base pair polyadenine repeat microsatellite sequence and up to 80% of colon cancer patients with HNPCC present mutated forms of T*β*RII [43]. To summarize, the specific response to TGF-*β* during tumor progression depends on the stage of carcinogenesis and the responsiveness of the tumor cells and can be attributed to both independent and interrelated factors including changes in (1) TGF-*β* expression; (2) TGF-*β* receptor expression; (3) availability of downstream signaling components; (4) evasion of the immune response; (5) stimulation of inflammation; (6) presence of local and systemic factors (autocrine, endocrine, paracrine, juxtacrine, or matricrine interactions); and (7) the recruitment of cell types that lead to advanced tumor growth or promote angiogenesis [26, 27].

## 4. Reactive Oxygen Species in Cancer 

Reactive Oxygen Species are a family of reactive molecules which are constantly generated in all aerobic species. Although ROS levels are balanced by well controlled rate of generation and elimination or consuming in normal cell conditions, in cancer cells where a dysregulated oxidative stress may exist, excessive ROS contribute to the chemical damage of proteins, lipids, and DNA [44] which all may contribute to tumorigenesis.

Chemically, ROS are small molecules derived from oxygen, comprising free radical and non-free radical oxygen intermediates, ions, or molecules that have a single unpaired electron in their outermost shell of electrons and are constantly generated inside cells by enzyme complexes or as by-products of redox reactions, including those underlying mitochondrial respiration [45]. ROS include oxygen radicals (superoxide anion (O_2_^•−^), hydroxyl (^•^OH), peroxyl (RO_2_^•^), and alkoxyl (RO^•^)) and certain nonradicals that either are oxidizing agents and/or are easily converted into radicals, such as hypochlorous acid (HOCl), ozone (O^3^), singlet oxygen (_1_O^2^), and hydrogen peroxide (H_2_O_2_). Nitrogen-containing oxidants, such as nitric oxide (^•^NO) and peroxynitrate (^•^NO_2_) are called reactive nitrogen species (RNS) (revised in [44] and references therein). The tumor-associated redox imbalance has been correlated with increased metabolic activity, mitochondrial dysfunction, deregulated cellular receptor signaling, peroxisome activity, oncogene activation, cyclooxygenases (COXes), lipoxygenases (LOXes), and thymidine phosphorylases, depending on the status of cancer cells and their cross-talk with stroma and infiltrating immune cells and enhanced activity of NADPH oxidase (Nox) [46, 47].

The increased generation of superoxide anion is mostly accomplished by the Nox/dual oxidase (Duox) family, whose prototypical member is the phagocytic Nox (Phox/Nox2) [46, 48]. Five forms of NADPH oxidase have been found: Nox1, Nox2, Nox3, Nox4, and Nox5 and two forms of dual oxidase: Duox1 and Duox2. These enzymes are all together now referred to as the NOX family [49]. The main characteristic shared by all NOX family members is that they are transmembrane proteins transporting electrons across biological membranes to produce superoxide by reducing oxygen. Nox family members also possess common conserved structural properties: (a) NADPH-binding site at the very COOH terminus; (b) an FAD-binding region in the proximity of the most COOH-terminal transmembrane domain; (c) six conserved transmembrane domains; and (d) four highly conserved heme-binding histidines, two in the third and two in the fifth transmembrane domain [49]. Importantly, the small GTPase Rac1 was shown to be a key molecular mediator of Nox1, -2, and -3, whereas Nox4, -5 and Doux seem to be independent of Rac1 [46, 49].

It has been proposed that, in tumorigenesis, ROS promote several aspects of tumor development and progression depending on mutagenic potential (initiation); regulation of intracellular signal pathways involved in the regulation of cell proliferation and survival; impact on cell motility, invasiveness, and metastasis; and cancer cells interplay with tumor stroma regulating inflammation responses, tissue repair, and angiogenesis which is vital for tumor growth and dissemination [47].

## 5. TGF-***β*** and ROS Interplay

### 5.1. ROS Activity Is Regulated by TGF-*β*

TGF-*β* is able to stimulate ROS production both in transformed and nontransformed cells [10] and several studies have shown that TGF-*β* can induce ROS production in different cellular compartments. TGF-*β* induces ROS production in mitochondria and microsomes in hepatocytes [50, 51], as well as mink lung epithelial cells by decreasing the activity of complex IV [52]. A mitochondrial thioredoxin (TXN2) sensitive mechanism which regulates the TGF-*β*-induced ROS production in mouse mammary epithelial cells has recently been described. These data indicate that a cysteine thiol-disulfide exchange reaction in mitochondria may be involved in TGF-*β*-mediated regulation of ROS and gene expression [13].

TGF-*β* also stimulates ROS production by activating H_2_O_2_-generating NADH oxidase in human lung fibroblasts [53]. TGF-*β* seems to activate Noxs via Rac1 dependent way [11]. It activates and/or induces Nox4 in several kinds of cells* in vivo* and* in vitro* [10]. The induction of* NOX4* gene expression by TGF-*β* is Smad3 dependent, and this effect is strongly counteracted by wild type p53 in breast cancer cells [54, 55]. In addition, TGF-*β* was shown to induce* NOX4 *gene expression along with ROS increase, while the downregulation of* NOX4* reduced ROS synthesis, indicating that Nox4 is one of the main sources of ROS in pancreatic cancer cells [56]. TGF-*β* can also induce* NOX2* gene expression and its activation dependent on p40phox subunit (NCF4) in adenocarcinoma Hela cells [57].

**Figure 2:**
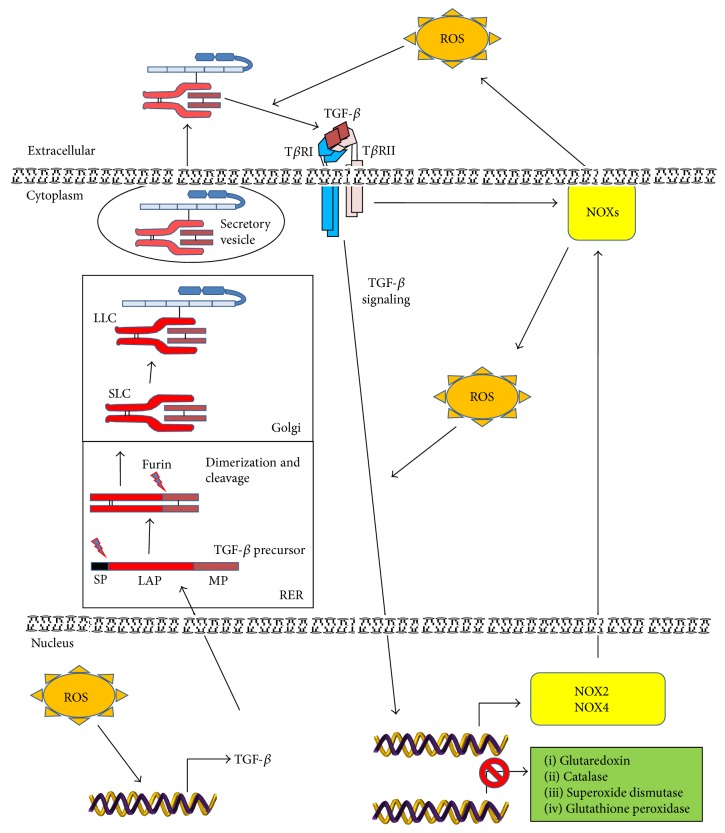
TGF-*β* and ROS interplay. TGF-*β* is synthesized as an inactive precursor protein. The signal peptide (SP), which leads the TGF-*β* precursor protein through its secretory pathway, is cleaved during the transit through the rough endoplasmic reticulum (RER), this way forming a protein homodimer. Its cleavage by furin convertase produces the small latent complex (SLC) in which mature TGF-*β* remains noncovalently bound to latency-associated peptide (LAP). Next, SLC by covalent binding to latent TGF-*β* binding protein (LTBP) produces the large latent complex (LLC). Finally, LLC is secreted and stored in the extracellular matrix for subsequent activation. The increased levels of ROS induce the release and bioavailability of TGF-*β* from the LLC through the oxidative modification of LAP. Active TGF-*β* by binding to its cell surface receptors induces the activation of downstream pathways, which further regulate ROS production by both NOX activation and increased NOX expression or by downregulation of antioxidative proteins expression. In addition, increased ROS production may directly induce TGF-*β* expression.

TGF-*β* regulates ROS activity, not only by inducing their production, but also by downregulating the expression of antioxidant enzymes such as glutaredoxin, catalase, superoxide dismutase (SOD), and glutathione peroxidase (GPx) [10]. Moreover, TGF-*β* can induce a decrease in concentration of the important antioxidant glutathione (GSH). One possible mechanism which can explain the mechanism by which TGF-*β* decreases GSH concentration involves regulated expression of the GSH catalytic subunit gamma-glutamylcysteine synthetase (GLC). TGF-*β* inhibits the expression of GLC, this way causing a dramatic reduction in both GLC activity and GSH levels in the adenocarcinomic human alveolar basal epithelial cells A549 [58]. The capacity of TGF-*β* to regulate GLC depends on the transactivation and binding of c-Jun and Fra-1 complex to AP-1 site in the GLC promoter, as well as the binding of Smad3 to GLC promoter at the same time increasing the expression of the transcription factor ATF3, this way producing a repression of GLC gene expression [10, 59, 60]. These data indicate that TGF-*β* is able to increase ROS levels by downregulating the expression of antioxidant enzymes concomitantly with the reduction of antioxidant compounds (Figure 2).

### 5.2. TGF-*β* Signaling Is Regulated by ROS

It is believed that ROS mediates the TGF-*β*-regulated expression of a number of genes, but little is known about how ROS may regulate the activation of TGF-*β* intracellular signal transduction. As we already mentioned, TGF-*β* is able to trigger many signaling pathways which include Smad and non-Smad mechanisms. Smad2 signaling seems to be sensitive to ROS effects, due to studies which showed that TGF-*β*-stimulated Smad2 phosphorylation can be inhibited by N-acetyl cysteine (NAC), reduced glutathione and L-cysteine [61]. These results suggested that thiol groups are important for the suppression of Smad2 activation and for the prevention of Smad2/Smad4 complexes accumulation in the nucleus [62, 63]. It has recently been reported that chronic exposure to H_2_O_2_ provokes a reduction in T*β*RII and Smad3 expression which impairs TGF-*β* signaling in human skin fibroblasts [64]. These results are interesting because they show that sustained ROS levels may act as a negative feedback on TGF-*β* signaling, implying the mechanism by which ROS may contribute to tumorigenesis. In support of our previous statement, some types of cancers show frequent reduction in T*β*RII expression [65].

ROS are able to modulate the TGF-*β*-mediated activation of MAPKs by indirect regulation of the activity of phosphatases which dephosphorylate tyrosine and/or serine/threonine groups. Protein tyrosine phosphatases such as PTP1B, serine/threonine phosphatases such as protein phosphatase 2A (PP2A), and some dual specific MAPK phosphatases (DS-MKPs) such as MKP-1 and MKP-3 can be inactivated by ROS through oxidation of critical cysteine residues in their active sites, associated with a sustained activation of MAPK [10, 66]. Additionally, TGF-*β* also activates NF-*κ*B in a ROS dependent way [11], as ROS can influence NF-*κ*B signaling by potentiating IKK/NEMO dimerization. IKK complex activation is probably mediated by ROS-sensitive IKK phosphatases. Activated IKK phosphorylates IKB inducing the release and activation of NF-*κ*B [67]. Intriguingly, oxidation of NF-*κ*B by ROS inhibits its DNA binding ability provoking a negative regulation at the nucleus compartment and inhibition of transcriptional activities of NF-*κ*B [68].

### 5.3. ROS Activate Latent TGF-*β*

TGF-*β* is firstly synthesized as an inactive multiprotein precursor complex consisting of a signal peptide, latency-associated peptide (LAP) domain and mature TGF-*β* (Figure 2). During its transit through the rough endoplasmic reticulum, the hydrophobic signal peptide is proteolytically cleaved from the inactive complex and a dimeric pro-TGF-*β* complex is formed. The furin-like convertase makes the second cleavage, producing the protein complex consisting of LAP and TGF-*β* mature protein. The noncovalent bonds between these components prevent the activation of the mature protein, thus creating a small latent complex (SLC), which passes through the Golgi apparatus. The SLC binds to a latent 125–160 kDa TGF-*β* binding protein (LTBP) via a disulphide bond giving rise to the large latent complex (LLC) [69]. Immediately after secretion this complex is sequestered by the ECM; hence TGF-*β* needs to be activated and released from ECM in order to exert its cellular effects [14]. The N-terminal region of LTBP is covalently cross-linked to the ECM by extracellular tissue transglutaminase. The hinge domain of LTBP is a protease-sensitive region and, thus, LLC can be released from the ECM by proteolytic cleavage. To become bioavailable and capable of binding to its cell surface receptor, TGF-*β* has to be dissociated from LAP in SLC and/or LLC [14].

Release of TGF-*β* from LAP, a process called latent TGF-*β* activation, is required for the binding of TGF-*β* to its receptors [10]. Extracellular activation of the latent TGF-*β* is a complex and important process in the regulation of TGF-*β* functions* in vivo*. The interaction between TGF-*β* and LAP is not covalent and can be disrupted by both proteolytic and nonproteolytic mechanisms. Physicochemical and biological variables that may participate in the regulation of TGF-*β* activation are heat, local acidification, thrombospondin-1 (TSP1), integrins, proteinases, and oxidative modification of LAP due to its exposure to ROS [69–74].

Barcellos-Hoff and Dix [73] demonstrated that exposure to ionizing radiation or metal iron plus ascorbate provokes TGF-*β* activation, possibly by oxidative-dependent conformation changes in the latent complex which allow the release of TGF-*β*. Similar experiments using asbestos and ascorbic acid also activated recombinant latent TGF-*β*, and the participation of ROS was demonstrated by addition of superoxide dismutase, catalase, or deferoxamine which significantly reduced TGF-*β* activation. The activation of latent TGF-*β* by asbestos-ascorbate-mediated generation of ROS apparently resulted from oxidative modification in LAP, leading to loss of its ability to bind to TGF-*β*1 [10, 75]. Although three isoforms of TGF-*β* have been discovered in mammals, only latent TGF-*β*1 complex was shown to be sensitive to redox-mediated activation. Site-specific mutation at methionine 253 in LAP/TGF-*β*1 was critical for the latent TGF-*β* activation by ROS, as this amino acid may act as a redox switch allowing latent TGF-*β*1 to act uniquely as an extracellular sensor of oxidative stress in tissues [10, 76]. The response of the TGF-*β* sensor to certain types of oxidative stress may reflect a need for cells to produce TGF-*β* during processes such as inflammation and apoptosis that can cause ECM damage through the production of ROS [14] and may act as protumorigenic signal in which ROS activation and release of TGF-*β* contribute to tumor progression.

Intriguingly, a regulatory loop between oxidative stress/ROS and TGF-*β* can be established in cancer cells. TGF-*β* regulates oxidative stress by both incrementing ROS production and regulating the antioxidative system. Meanwhile, ROS may regulate Smads signaling which contributes to cancer cells' resistance to proliferation inhibition by TGF-*β* [77] in the early stage of tumorigenesis, contrary to the enhancement of MAPK and NF-*κ*B pathways. Moreover, ROS may regulate TGF-*β* expression in epithelial cells [78] and concomitantly participate in the activation of latent TGF-*β* complex in the ECM, this way incrementing TGF-*β* bioavailability. Thus, this ROS-TGF-*β* interplay strongly contributes to tumorigenesis, avoiding inhibition of cell proliferation and incrementing cancer cell malignancy. Moreover, TGF-*β* is involved in multiple redox-regulated signaling pathways in cancer by regulating redox-sensitive transcription factors and signaling molecules. The increase of ROS may contribute to increased genomic mutation rates during cancer initiation [79]. Thus, ROS can convert the antitumorigenic role of TGF-*β* in the early stage of tumor progression to a protumorigenic role and this ROS-TGF-*β* interplay may perpetuate the cancer phenotype.

## 6. TGF-***β*** and ROS in Epithelial Mesenchymal Transition

EMT is a differentiation process by which epithelial cells undergo transition to mesenchymal cells. It occurs during embryogenesis and tissue morphogenesis (type 1 EMT); wound healing and tissue fibrosis (type 2 EMT); and cancer progression (type 3 EMT) [27, 80, 81]. Many types of cancer cells depend on EMT to obtain a migratory phenotype which enables them to leave primary carcinomas and invade other tissues [5, 82]. During EMT, early phenotypic changes involve loss of epithelial cell-cell contacts by downregulation of junction complex members, which include typical epithelial markers, claudin-1, ZO-1, and E-cadherin. Interestingly, as E-cadherin plays a critical role in the epithelial homeostasis, its downregulation can lead to decreased expression and/or organization of additional epithelial markers, desmosomal proteins (such as plakoglobin, desmogleins, and desmoplakins) [82, 83]. Furthermore, epithelial cells undergo an array of modifications during EMT: they lose the apical-basal polarity showing spindle cell phenotype; their cytoskeleton is subjected to profound reorganization; the expression of cytokeratins is lost, along with the expression of mesenchymal vimentin network and rearrangement of actin cytoskeleton. Together with an increase of motile behavior, all these events cooperate to increase tumor cell motility and invasive cell phenotype [84–86].

Currently, TGF-*β* is recognized as a master regulator of EMT, since it participates in all types of the mentioned differentiation processes. Tumor cells persistently exposed to TGF-*β* elicit EMT, which plays a pivotal role in cancer progression [27, 80]. In type 3 EMT, TGF-*β* may cooperate with several other oncogenic pathways to induce and maintain the mesenchymal phenotype of metastatic tumor cells, allowing the regulation of TGF-*β*-induced genes and downregulation of E-cadherin expression among others [85, 87].

TGF-*β* can induce EMT by activating Smad3 signaling, which, together with Smad4, has been shown to be crucial in EMT promotion [25, 84, 88, 89]. In contrast to the role of Smad3, Smad2 has been postulated as an inhibitor of EMT, since Smad2 ablation enhances EMT during skin carcinogenesis [90]. Conversely, Smad2 has also been shown to participate in the TGF-*β*1-induced EMT, since overexpression of constitutively active Smad2 enhances EMT in carcinoma cells in cooperation with H-Ras oncogene [91]. Considering disparate results, further analyses are necessary to elucidate the specific role of Smad2 in EMT. The capacity of TGF-*β* to induce EMT also requires cooperation with a number of different intracellular signaling pathways, such as Ras and Rho GTPases (Rho and Rac1), MAPKs, Wnts, and NF-*κ*B [11, 92–94]. TGF-*β* regulates the expression of EMT-involved genes by modulating the expression of transcription factors such as Snail and Slug (corresponding human genes are named SNAI1 and SNAI2, resp.). For example, Snail mediates TGF-*β*-induced EMT by repressing E-cadherin transcription and stimulating the expression of mesenchymal genes, vimentin and *α*-SMA, among others. In turn, Snail promotes collagen-I synthesis and deposition and may upregulate the expression of proinflammatory interleukins IL-1, -6, and -8 which produce an inflammatory microenvironment supporting the acquisition of EMT of the cancer cells [85, 95–97]. During EMT cells acquire mesenchymal and stem cell-like features, increasing their motility and invasiveness, as well as becoming resistant to apoptosis and acquiring anchorage-independent growth. Furthermore, upregulation of serine proteinases such as urokinase plasminogen activator (uPA) and matrix metalloproteinases (MMPs) leads to the degradation of ECM proteins and provides tumor cells additional mechanisms to invade surrounding tissues and colonize distant organs [27, 28, 98, 99].

The influence of ROS on EMT in cancer cells has been well documented [12]. One mechanism by which ROS can induce EMT is by its interplay with Snail. ROS can activate Snail and increased levels of Snail induce intracellular ROS levels, this way creating a self-regulating loop which leads to EMT [12, 100, 101]. Hypoxia has also been implicated in ROS-Snail interaction during EMT, as ROS are not only produced by aberrant function of mitochondrial complex III during hypoxic stress but also stabilized by hypoxia inducible factor 1 (HIF-1) [12]. Moreover, HIF-1 induces Snail expression [102] indicating that hypoxia can provoke a positive scenario for ROS-Snail interplay in order to enhance EMT in cancer cells. Interestingly, increased ROS levels cause intercellular dissociation by provoking a tyrosine phosphorylation of p120 catenin and its cytoplasmic translocation along with intense cytoskeleton reorganization [103].

Mounting evidence also suggests that TGF-*β*-induced EMT may be mediated by ROS induction [104]. As a multipotent cytokine, TGF-*β* also increases the production of extracellular matrix proteinases; cell motility; and invasiveness, which all together collaborate to enhance tumor progression [20, 27, 99]. In order to induce EMT, TGF-*β* can increase ROS by two mechanisms: (1) through the inhibition of the antioxidative capacity of cancer cells and (2) through direct regulation of ROS production. The inhibition of antioxidative capacity of cancer cells by TGF-*β* is established through the inhibited expression of antioxidative enzymes. One such enzyme is cytosolic dithiol glutaredoxin (Grx1) [105], member of oxidoreductases thioredoxin superfamily, which mediates the reversible electron transfer in reduced/oxidized glutathione (GSH/GSSG) [106]. It was shown that TGF-*β*, in EpRas mouse mammary epithelial cells, downregulated Grx1 expression and increased ROS levels concomitantly with EMT development. These effects were reverted by exogenous expression of Grx1 as well as treatment with NAC, suggesting that the decrease of intracellular antioxidant mechanism is critical for TGF-*β*-induced EMT [107]. TGF-*β*-mediated EMT was also inhibited by exogenously expressed mitochondrial thioredoxin (TXN2), independently of Smad signaling. Since TXN2 is antioxidant acting in a particular manner that facilitates the reduction of disulfide bonds on proteins by cysteine thiol-disulfide exchange, this implied that thiol oxidation can be a regulatory mechanism in TGF-*β*-mediated gene expression regulation associated with EMT [13]. In alveolar epithelial cells, the replenishment of intracellular GSH by NAC treatment was sufficient to block the capacity of TGF-*β* to induce EMT [108]. TGF-*β* highly reduces GSH in these cells, while NAC reduces ROS levels and increases GSH. Meanwhile, in the same cell model, H_2_O_2_ treatment did not induce EMT indicating that oxidative stress is necessary but not sufficient to induce EMT in these cells. Interestingly, NAC inhibits Smad3 activation by TGF-*β*, a pathway important for TGF-*β*-stimulated EMT [108].

As previously mentioned, the capacity of TGF-*β* to induce EMT is also mediated by its capacity to increase ROS production. Tobar et al. [11] demonstrated that TGF-*β* induces ROS by a mechanism dependent of Rac1/NOXs in mouse keratinocytes. ROS can further mediate NF-*κ*B activation and both collaborate to stimulate EMT induced by TGF-*β*. In addition, this effect was parallel with increased cell migration and extracellular matrix proteinases expression. Furthermore, ROS can induce TGF-*β* expression during EMT induction in human keratinocytes [109], suggesting the possibility of a TGF-*β*/ROS/TGF-*β* loop operating to induce EMT.

It has recently been reported that TGF-*β* increases* NOX4* gene expression by Smad3-dependent mechanism and that Nox4 contributes to ROS production which may be critical for the progression of the TGF-*β*-induced EMT in breast cancer and moreover indicates the role of Nox4 in the enhancement of cell motility, without affecting cell proliferation [52]. The role of Nox4 has also been confirmed in pancreatic cancer cells, where blocking of Nox4 impaired TGF-*β*-induced EMT. Also, the Nox4-induced ROS partly regulated the activity of tyrosine phosphatase-1B (PTP1B), a well-established redox sensor that can mediate ROS-induced intracellular signaling [110].

In addition, TGF-*β* can induce the expression of Nox2 during EMT and this activation was dependent on p40phox subunit (NCF4) which regulates the expression of other cytosolic regulatory components, including p47phox, p67phox. Interestingly, NCF4 overexpression was sufficient to regulate key markers of expression involved in EMT such as Snail, Slug, and E-cadherin. Moreover, NCF4 seemed to mediate the transcription/translation regulatory Y-box binding protein-1 (YB-1) expression by TGF-*β*. YB-1 is a broad-specificity RNA-binding protein, mainly involved in the regulation of mRNA transcription, splicing, translation, and stability and has recently been implicated in cancer progression and EMT [57].

**Figure 3:**
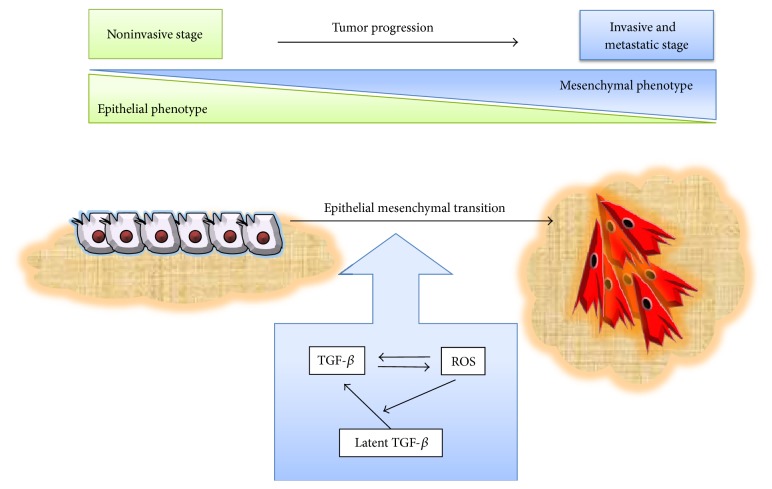
TGF-*β* and ROS cooperate in the induction of epithelial mesenchymal transition. Both TGF-*β* and ROS are involved in the induction of EMT, as well as their mutual cooperation. TGF-*β* stimulates ROS production in cancer cells, and the enhancement of ROS levels in turn may induce the activation of extracellular matrix-associated TGF-*β* latent complex, thus exacerbating TGF-*β*-induced EMT. Meanwhile, the increment of ROS also stimulates EMT, and both finally may collaborate to induce conversion of the epithelial to the mesenchymal phenotype, thus strengthening tumor progression and metastasis.

The prostate transmembrane protein androgen induced-1 (TMEPAI) has been suggested as a novel mediator affecting the capacity of ROS to act on TGF-*β*-induced EMT. TEMPAI is able to antagonize TGF-*β* signaling by interfering with T*β*RI/ALK5-induced R-Smads phosphorylation and is overexpressed in epithelial cancer and highly susceptible to induction by TGF-*β* [111, 112]. Experiments conducted in A549 cells showed that TMEPAI mediates TGF-*β*-induced ROS production and EMT, as the knockdown of TEMPAI dramatically reduced ROS concomitantly with decreased EMT. Moreover, TMEPAI is able to downregulate insulin receptor substrate-1 (IRS-1), an EMT suppressor which plays an important role in maintaining the epithelial phenotype in cancer cells [113], via ROS [114]. Clearly, TGF-*β* and ROS together participate in the induction of EMT, as several studies demonstrated that antioxidant treatments are sufficient to reduce the capacity of TGF-*β* to induce EMT in cancer cells, strongly suggesting the critical role of oxidative stress in this process. Moreover, ROS production, dependent or not on TGF-*β*, may induce TGF-*β* expression [10, 78] which may contribute to the development of EMT by producing a positive ROS-TGF-*β* feedback in cancer cells (Figure 3).

It is believed that EMT acts as a driving force in tumor progression; it enables cancer cells to invade surrounding tissues and colonize remote sites. Indeed, EMT represents one of the main steps in the acquisition of migratory phenotype of cancer cells and greatly collaborates in the pathogenesis of cancer [115]. The redox control of EMT emerges as an important factor which increases tumor malignancy; thus a prooxidant environment in the tumor, acting on both cancer and stroma-associated cells, may accelerate tumor progression [47]. In addition, excess of ROS is deleterious to normal cells, while the persistent prooxidative state in cancer cells can lead to intrinsic oxidative stress [79]. Oxidative stress/ROS may modify cellular response to TGF-*β* either by inducing genetic changes or by regulating cellular behavior, changing the role of TGF-*β* from tumor inhibiting factor to tumor promoting, and furthermore collaborating with TGF-*β* to lead to EMT in cancer cells with the consequences in tumor progression. Interestingly, in the tumor microenvironment, common sources of TGF-*β* are cancer and stromal cells, such as infiltrating immune cells and fibroblast (revised in [116]). This TGF-*β* expression may be regulated by ROS production within the tumor concomitantly with Smad and non-Smad signaling pathway modulation, finally leading to EMT [63]. One of the crucial events in cancer disease is the metastasis formation, which is considered a complex multistep event with sequential molecular and cellular events including EMT, migration and invasion, blood vessel intra- and extravasation, survival, and growth in a new tissue environment [79], in which the mutual interaction of oxidative stress/ROS and TGF-*β* may play a role in almost all stages described. In addition, both ROS and TGF-*β* also have important roles in innate immune system response [117], allowing cancer cells to escape from immune surveillance and incrementing tumor growth and development, which together with the induction of EMT strongly cooperate to enhance tumor progression which is deleterious to cancer patient survival.

## 7. ROS and TGF-***β*** in Cellular Senescence and Cancer

It is believed that oxidative stress/ROS are important contributors in determining cellular senescence in mammalian cells [118–120]. Increased levels of ROS may result in macromolecular damage (proteins, lipids, and nucleic acids) and are involved in important mechanisms responsible for cellular senescence, aging and in the development of several age-associated diseases [15]. ROS can induce cellular senescence by a telomere-dependent mechanism (revised in [121]) and telomere-independent mechanism [122] involving unrepairable single or double-strand DNA breaks. A major DNA lesion generated by excessive ROS is 8-oxo-2′-deoxyguanosine, which is accumulated in senescing human cell cultures and in ageing mice. The most important effects resulting from DNA damage are genomic instability and mutations, which can lead to the development of tumors. Normally, DNA damage is removed by well functioning repair systems which prevent harmful cellular changes. However, with increasing age, these systems are more weakened, contributing to genetic lesions and increased number of cells damaged, with unfortunate consequences on the development of age-specific diseases including arthrosclerosis, neurodegenerative diseases, and cancer [123]. Specifically, ROS are known to induce genomic alterations such as point mutations and deletions and inhibit tumor suppressor genes as well as induce the expression of oncogenes. Moreover, ROS may reversibly regulate intracellular signaling targets such as MAPK, PKC, PI3K, and phosphatases, resulting in the reorganization of cytoskeleton, adhesion, and cell migration [119], which collaborate in the enhancement of cell malignancy.

Intriguingly, senescence protects damaged cells from neoplastic transformation by inducing a stable growth arrest, which is parallel with a secretion of a complex mixture of factors named as senescence-associated secretory phenotype (SASP). SAPS may mediate tumor suppressive effects but also in another way may exert a protumorigenic role, potentiating cancer cell malignancy [124].

Essential roles of TGF-*β*, contributing to its capacity to suppress early stages of epithelial tumorigenesis, involve induction of cell arrest, stimulation of apoptosis, and promotion of genomic stability and cellular senescence [2]. It has been reported in human lung adenocarcinoma cell line that TGF-*β*, like ROS, may trigger two independent senescence programs: one dependent on telomere shortening and second premature senescence independent of telomere shortening. When these cells were driven to a senescence state, they secreted the senescence-associated cytokine IL-6 without tumorigenic capacities in nude mice, suggesting that TGF-*β*-forced induction of senescence in cancer cells can be a potential anticancer therapy [125].

One of the main mechanisms by which TGF-*β* acts as a negative regulator of cell cycle and as a tumor suppressor is its capacity to induce cyclin-dependent kinases inhibitors p15 (ink4b), p16 (ink4a), p21 (Waf/Cip1), and p57. In primary mouse epidermal keratinocytes engineered to express a viral Ha-Ras oncogene, which have a high proliferation rate, TGF-*β* strongly induced growth arrest, and senescence associated with the expression of p16 and p19 (ARF). Expression of these kinases was dependent on Smad3 signaling. Moreover, genetic deletion of the cdkn2a (ink4a/arf) locus reduced cell sensitivity to TGF-*β* mediated cell cycle arrest and induction of senescence. This supported the idea that alteration of TGF-*β* molecular and cellular response may be influenced by cdkn2a locus inactivation during tumor development [77]. It is believed that the induction of senescence is an attractive mechanism for the treatments of some types of cancers. In that way, selected hepatocellular carcinoma (HCC) cells with intact Smad signaling were highly responsive to TGF-*β*-induced cell arrest and senescence both* in vitro* and* in vivo*, with increased expression of p15 (ink4b) and p21 (Waf/Cip1). In contrast, the inhibition of TGF-*β* by T*β*RII deletion abolished* in vitro* senescence response and greatly accelerated* in vivo* tumor growth. Interestingly, the induction of senescence was also associated with the TGF-*β*-mediated induction of Nox4 expression and ROS production. The reduction of ROS by using NAC or Nox4 siRNA inhibited TGF-*β*-induced senescence and accumulation of p15 and p21 [126]. It has also been suggested that TGF-*β* may participate in oxidative stress-induced senescence by regulating the expression of adenine nucleotide translocase-2 (ANT2). ANT2 is consistently downregulated during the course of all major types of cellular senescence including replicative, oncogene- and radiation/drug-induced forms in normal human diploid fibroblasts. TGF-*β*, by inducing formation of NF1/Smad4 complex, inhibits ANT2 in cancer cells, and this repression of ANT2 contributes to senescence-associated oxidative stress and DNA damage [127].

As mentioned previously, several cancers showed reduced response to TGF-*β* by presenting abnormalities in TGF-*β* receptors or Smad4, a mechanism by which cancer cells may escape from TGF-*β*-induced senescence. In viral Ha-Ras keratinocytes TGF-*β*-null and dominant negative T*β*RII transfected cells are highly resistant to cell cycle arrest and senescence. These cells show low expression of p15 (ink4b) and p16 (ink4a) and high levels of cdk4 and cdk2 activity. Therefore, inactivation of TGF-*β*1 expression or its response seems to be sufficient to overcome the senescence program and accelerate malignant progression [128]. Similarly, the expression of a dominant-negative T*β*RII abrogated autocrine TGF-*β* signaling in telomerase-immortalized HMECs and suppressed H-Ras-V12-induced senescence-like growth arrest [129].

It has been suggested that cancer cells can also induce a form of “accelerated aging” in cancer-associated fibroblasts, via ROS production and oxidative stress. This in turn may provide a more supportive microenvironment to tumor cells growth [130]. In this aspect, it has recently been reported that, in genetically unstable oral squamous cell carcinomas (GU-OSCC), fibroblasts are displaying senescent phenotype. The increased levels of ROS in tumors induced fibroblast-dependent TGF-*β* production, and this factor induced fibroblast senescence. Moreover, senescent fibroblasts show high capacity to stimulate malignant keratinocytes invasion* in vitro*. This study demonstrated that, in GU-OSCC, the senescent cancer-associated fibroblasts, maintained by ROS and TGF-*β* induced by cancer cells, promote tumor malignancy [16].

## 8. Concluding Remarks

A large number of evidence in the literature confirms the important role of TGF-*β* and ROS in the course of cancer progression and metastasis. Due to its importance in tumorigenesis, TGF-*β* and oxidative stress/ROS system is a very attractive target in cancer chemotherapy.

Targeting TGF-*β* has already been clinically tested in therapeutic approaches. These strategies included small inhibitors of the enzymatic activity of uPA or TGF-*β* receptors, specific neutralizing antibodies, peptide inhibitors such as p44, and therapeutic approaches to control the expression of TGF-*β* signaling components at transcriptional level, among others [17]. Currently, most clinical trials have failed to show beneficial effects of dietary antioxidants in a variety of cancer types [9, 131]. ROS can have contrary roles in tumorigenesis, as they can trigger aberrant procancer signaling and DNA mutations, whereas high levels of ROS can be toxic to cancer cells, this way affecting their survival. Thus, ROS status seems to be a critical aspect in the balance between cancer cell survival and their advance to more malignant stages and tumor suppression and cell death, which can easily be disrupted in favor of each side. In one way, cancer cells, by increasing their antioxidant capacity, may balance oxidative stress status, suggesting that prooxidant approaches may be exploited in cancer therapy. Some evidence are indicating that dietary antioxidants contribute to tumorigenesis, probably by protecting cancer cells from ROS-induced cell death [132]. One of the most fascinating aspects of cancer cells is their capacity to adapt and reprogram their homeostasis to oncogene mutations, changes in metabolism, extreme changes in microenvironment (including hypoxia and acidic changes), taking advantage of these stressors to survive and increase cell malignance [9]. In fact, ROS in more advanced tumor stages may collaborate with growth factors (TGF-*β*) to increase cell survival, migration and invasion, and finally metastasis. Thus, cancer cells can balance prooxidant and antioxidant activities, in a well orchestrated regulation, by taking advantage from the changes in ROS homeostasis in order to survive and to progress in tumorigenesis.

In this review, we attempted to reveal the interplay between TGF-*β* and ROS. We believe that the inhibition or regulation of the amplification loop operated between TGF-*β* and ROS system in tumor cells could limit tumor progression and metastasis, impairing tumor dissemination, proliferation, and survival. We hope future clinical trials using combined therapies which target TGF-*β* and oxidative stress/ROS could increase the success of cancer treatment. Moreover, TGF-*β* and ROS induce EMT, which enhances tumor cells migration and invasion. In addition, TGF-*β* and ROS are both implicated in cellular senescence, which can be a useful therapy target in the early stages of tumor progression. This also opens the question about the use of antioxidants in oncotherapies, since they may delay epithelial cancer cells senescence dependent on the ROS increment. However, the use of antioxidants in combination with current clinical treatments should be carefully considered, since antioxidants can determine the senescence of fibroblasts in tumor stroma, this way enhancing tumorigenesis. By regulating TGF-*β* and oxidative stress/ROS it could be possible to control the positive tumor microenvironment and cancer-stroma cells interactions.

Finally, elucidating the complex interplay and roles of TGF-*β* and oxidative stress in cancer is critical for the understanding of their participation in the initiation, progression, and tumor metastasis and could eventually uncover potential combinatory therapeutics for future cancer treatment in humans.
